# Secondary 1 Program of Project P.A.T.H.S.: Process Evaluation Based on the Co-Walker Scheme

**DOI:** 10.1100/tsw.2009.60

**Published:** 2009-08-01

**Authors:** Daniel T. L. Shek, Catalina S. M. Ng

**Affiliations:** ^1^ Department of Applied Social Sciences, Hong Kong Polytechnic University, Hong Kong, China; ^2^ Department of Sociology, East China Normal University, Shanghai, China; ^3^ Kiang Wu Nursing College of Macau, Macau

**Keywords:** co-walker scheme, observation, positive youth development program, process evaluation

## Abstract

This study examined the implementation quality of the Tier 1 Program (Secondary 1 Curriculum) delivered in the second year of the Full Implementation Phase of the Project P.A.T.H.S. (Positive Adolescent Training through Holistic Social Programmes). Under the “Co-Walker Scheme”, systematic observation of curriculum units was conducted in 138 schools. Results indicated that the overall level of program adherence was high, with an average of 82.9%. The mean ratings of the program implementation quality were also high. Despite limitations, the findings of this study suggest that the implementation of the Secondary 1 Program (Tier 1 Program) of the Full Implementation Phase was of very high quality. The present findings also provide strong evidence to account for the successful and encouraging outcomes of a major positive youth development program in Hong Kong.

## INTRODUCTION

Youth development programs are gaining momentum as a promising approach for the prevention of risk behavior among adolescents. Despite the reported promise and growing popularity of this approach in Western countries, much remains to be done in non-Western contexts such as Hong Kong, which is an international city with a strong Chinese tradition. Unfortunately, there are very few systematic and multiyear positive youth development programs in Hong Kong. Even if such programs exist, they are limited in scope as they primarily focus on isolated problems and issues in youth development (i.e., deficits-oriented programs). Furthermore, most notably, youth development programs in Hong Kong are relatively short-term in nature[1], and systematic and long-term evaluation of the available programs is lacking. Against this background, primary prevention programs that target specific adolescent developmental problems and positive youth development programs are badly needed in Hong Kong[2] because consistent findings from both published and unpublished studies demonstrate that Hong Kong teenagers face a number of developmental problems, such as mental health problems like depression[3], abuse of psychotropic substances[4], adolescent suicide[5], deliberate self-harm[6], school violence[7], and erosion of family solidarity[3].

Great strides have been made in the recent decade to promote holistic development among adolescents in Hong Kong. Particularly promising is the territory-wide project entitled P.A.T.H.S. to Adulthood: A Jockey Club Youth Enhancement Scheme initiated by The Hong Kong Jockey Club Charities Trust. P.A.T.H.S. denotes **P**ositive **A**dolescent **T**raining through **H**olistic **S**ocial Programmes, with two tiers of programs. While the Tier 1 Program is a universal program that utilizes a curricula-based approach for all Secondary 1 to 3 students of the participating schools, the Tier 2 Program is provided for students who have greater psychosocial needs. The Trust invited academics of five local universities to form a research team, with the first author as the Principal Investigator, to develop a multiyear universal positive youth development program to promote holistic adolescent development.

Given the novelty of the project, numerous training workshops have been organized for instructors (e.g., teachers, social workers) who implement the Tier 1 Program so as to help them familiarize themselves with the program philosophy and content, the necessary teaching strategies, as well as the need for fidelity, and to cultivate their enthusiasm and support for the program[8,9]. Added to this, with the primary goals of providing both instrumental and emotional support to program implementers and to enhance the communication between the schools and the research team, the "Co-Walker Scheme" was launched. The term "co-walker" was deliberately coined to correspond to the Chinese translation "tong xing zhe", with "co-" meaning together and "walker" denoting a supporter. The entire term signifies unfailing collegial support.

In Hong Kong, the teaching profession is highly stressful[10,11,12] and teacher burnout is well documented[12,13]. The stress sources due to the nature of the job itself may come from the curriculum demands, the daily teaching, other duty commitments, and change in the ecology of the education sector because of the education reform since the handover of Hong Kong to China. Obviously, if teacher stress is left unresolved, it will have significant adverse impacts on teachers' physical and mental health. As recommended by Jin et al.[12], there is a desperate need to nurture a culture of collegial support for managing stress in the teaching environment.

Seeking social support is the predominant approach to cope with stress and prevent burnout[14]. Individuals may be said to have social support if they have a relationship with one or more people that is characterized by relatively frequent interactions, positive feelings, and perceived willingness to give emotional and/or instrumental assistance in times of need[15]. Apart from families, sources of support include coworkers, friends, spouses, and supervisors[16]. Folkman and Lazarus[17] assert that social support simultaneously can function as a problem-focused and an emotion-focused coping strategy. For example, talking to someone about a stressful event can function as a problem-focused coping strategy when the individual receives tangible information that helps to tackle the problem. Concurrently, the strategy of talking to someone can also function as an emotion-focused coping strategy when the emotional support given helps to regulate emotional responses arising from the stressful event. Hence, social support is conceptualized as a type of coping strategy[18].

Under the "Co-Walker Scheme", the prime objective is to offer workplace social support so that it enhances an individual's sense of personal competence in managing his/her workloads, which in turn may reduce his/her experience of exhaustion[19]. Workplace social support may be a particularly relevant buffer of work stress, as individuals spend substantial time at work. In substantiation of the importance of workplace social support, prior research reported that low coworker support is a risk factor for poor health, such as neck pain[20] and elevated cardiovascular activity[21]. On the other hand, social support is found to be associated with enhanced psychological well-being in the workplace[22], as it weakens the relationships between stressors and strains[23], and is an important resource for an individual to cope with stress and prevent burnout[14]. Reviews of the existing literature on teacher stress demonstrate that continued support from fellow colleagues fosters a teacher's ability to be resilient[24], and teachers experiencing less stress and burnout are those who work in a supportive environment[13].

Due to concerns about the mental health of the instructors, coupled with the fact that they are one of the key determinants of the program's success[25], as well as a key stakeholder, a trained colleague (i.e.,

"co-walker") from the Project P.A.T.H.S. is assigned to each participating school. The "co-walker" keeps regular contact with the school coordinator(s) via e-mails, phone calls, or mails so as to give advice and support to them, and pays at least one visit to each form of the school in every academic year. During the visit, the "co-walker" discusses with the instructors about the problems encountered and observes the implementation of the program in class. However, the primary purpose of the visit is to provide collegial support to the instructors and, so, the class observation involves no monitoring or purposes of evaluation. In sum, the "co-walker" functions as a resource person who gives instrumental support and, simultaneously, emotional support to the instructors.

Since the "Co-Walker Scheme" provides the program developers a unique opportunity to observe and study how the program was implemented in a naturalistic setting (i.e., classroom), the rich data complement and extend the existing literature. This is important because the findings on program implementation quality are under-reported in the prevention research literature despite its increasing importance[26]. For instance, in a review of over 1,200 published prevention studies, Durlak[27] found that less than 5% provided data on program implementation. In a meta-analysis of evaluation studies of primary and early secondary prevention programs published between 1980 and 1994, Dane and Schneider[26] reported that only 39 out of 162 evaluation studies documented procedures of fidelity. As information regarding factors that lead to successful or unsuccessful program implementation is particularly lacking[8], one of the primary purposes of the current study is to fill the knowledge gap of the program implementation in a non-Western setting.

To determine the effectiveness of an adolescent prevention program, Nation et al.[28] stressed that there are many factors that increase or decrease the likelihood of success of a program. Among these factors, process variables, such as diverse teaching methods (i.e., use of a wide range of teaching methods that help the program participants to become aware of and understand problem behaviors and acquire the related psychosocial skills) and positive relationships with adults (e.g., worker) are crucial factors. Furthermore, teaching practices and program implementation attributes also influence a program's success. For example, Tobler et al.[29] investigated what types of program were most effective in reducing, delaying, or preventing marijuana use, and examined whether the characteristics of the participants and program implementation factors were related to program success. Findings indicated that programs with high peer interaction were more effective than those with low peer interaction and that the delivery method instead of the program content determined the success of the program.

Although factors that significantly contribute to program implementation quality have been identified in previous studies, little has been documented on the inter-relationship among multiple facets of implementation[30]. To fill the existing research gap, the current study also explores the quality of program implementation with reference to the 5"P"s (i.e., program, people, process, policy, and place)[25].

First and foremost, program content (i.e., "program") focused on practical knowledge, competencies, and generic skills, and program design that was evidence based was conducive to the instructors' willingness to implement the program as intended, which in turn contributed to effective program implementation[31]. On the other hand, program activities that overlapped with other existing programs in the school were more likely to bore students, which adversely affected the program implementation[8]. In short, programs with interesting and developmentally appropriate activities that can capture the students' attention are preferable.

Another factor that significantly contributes to the effectiveness of school-based prevention programs is "people", such as principals, coordinators, and teachers[8]. The principal's leadership was found to be a vital factor influencing program success[32]. Additionally, committed coordinators who offered continued support to the front-line teachers fostered communication among different parties and provided clear guidance, and information pertaining to the program implementation contributed significantly to effective program implementation[8]. Prior studies further demonstrated that characteristics of the teachers, including extensive teaching experience, sufficient lesson preparation, high academic qualification like possession of a graduate degree, and a strong sense of belonging to the school, contributed to program effectiveness[31].

Regarding the program implementation process (i.e., "process"), effective delivery, such as using interactive teaching strategies[31] and facilitation skills to teach the program concepts and skills, and to generalize the students' learning across the school day[30], greatly contributed to the success of school-based prevention programs. Additionally, adherence to the designed program as well as good classroom and time management skills were salient process factors of quality implementation[8]. Kam et al.[30] stressed that continued support from the principal in the implementation process was vital because it encouraged teachers to devote themselves to the program implementation. Consequently, teachers who were more dedicated to teach were likely to create a positive classroom atmosphere, and to increase the learning interest and motivation of students[8].

Concerning the school policy (i.e., "policy"), schools that integrated the program into their formal curriculum and emphasized program fidelity were found to have effective program implementation[8]. The formulation of clear objectives in the program implementation process provided a clear direction for the program implementation[32]. Furthermore, good manpower deployment with division of labor was vital to prevent teacher burnout due to overloaded teaching and administrative duties[32]. Researchers suggested that it is important for schools to arrange regular meetings with the teachers to discuss problems encountered and the respective solutions, to build a supportive working environment, as well as to organize final evaluation meetings to applaud good work done, evaluate past practices, and obtain feedback[8].

Finally, an appropriate school environment with adequate facilities and instructional environment ("place") is conducive to effective program implementation. A nonauthoritarian working environment that demonstrates collaborative decision making and a positive atmosphere that enables job satisfaction facilitates effective program implementation[33]. Eccles and Gootman[34] further added several features that promoted positive youth development, including settings characterized with physical and psychological safety, appropriate structure, supportive relationships, opportunities to belong, positive social norms, support for efficacy, opportunities for skill building, and integration of family, school, and community efforts.

Although the effectiveness of the Tier 1 Program of the Project P.A.T.H.S., both in the EIP (Experimental Implementation Phase) (EIP-S1: 2005/06; EIP-S2: 2006/07; EIP-S3: 2007/08) and FIP (Full Implementation Phase) (FIP-S1: 2006/07; FIP-S2: 2007/08), was supported by several lines of evidence, such as objective outcome evaluation, subjective outcome evaluation[35,36], interim evaluation[37,38], and process evaluation[39], it is imperative to examine whether the implementation of the program is consistent with the program philosophy and design.

The primary purpose of this study was to examine the implementation quality of the Tier 1 Program (Secondary 1 Curriculum) based on systematic observation for the second year of the FIP (FIP-S1: 2007/08) and based on the data collected via the "Co-Walker Scheme". Based on the findings, implications for future program implementation are highlighted.

## METHODS

### Participants

A total of 213 schools participated in the Secondary 1 Curriculum of the Project P.A.T.H.S. in the Full Implementation Phase (FIP) in the 2007/08 academic year (FIP-S1: 2007/08). Among these schools, 105 schools adopted the full program (i.e., 20-h program involving 40 teaching units) and 108 schools joined the core program (i.e., 10-h program involving 20 teaching units). Among these participating schools, 138 schools agreed to have the lessons observed, with a total of 221 units observed. The average number of students per class was 35.57 and the average number of instructors per class was 1.88. The average duration of observation of one teaching unit was 36.57 min.

## Procedures

For each school participating in the "Co-Walker Scheme", systematic observations of one or two teaching units were conducted. There were 221 units under observation, which covered 14 positive youth development constructs encompassing bonding (BO), social competence (SC), emotional competence (EC), cognitive competence (CC), behavioral competence (BC), moral competence (MC), self-efficacy (SE), prosocial norms (PN), resilience (RE), self-determination (SD), spirituality (SP), clear and positive identity (ID), beliefs in the future (BF), and prosocial involvement (PI). Prior consent was sought from the principal before the observation was initiated. Informed consent was simultaneously obtained from the instructors (i.e., teachers and social workers) before the class observation took place. The prime objective of the visit (i.e., to provide collegial support) was repeatedly emphasized. The observers (i.e., the "co-walkers") were six colleagues of the project; all six were registered social workers. Nonetheless, as the fundamental aims of the "Co-Walker Scheme" are to give support to the instructors by sharing how the units are implemented and discussing the obstacles encountered, as well as to keep an administrative record, only one observer observed how the units were implemented.

While observing the class, the observer was required to fill in a form that covers four major areas:

- Basic information of the class observation (e.g., name of the school, number of students, number of instructor(s), sex of instructor(s), date of observation, and duration of the class period)
- Integration with school's formal curriculum
- Program fidelity and adherence
- Quality of program delivery, which included student interest, student participation and involvement, classroom control, interactive delivery method, strategies to enhance student motivation, use of positive and supportive feedback, instructors' familiarity with the students, opportunity for reflection, degree of achievement of the objectives, time management, lesson preparation, overall implementation quality, and success of implementation

In addition, the observer recorded his/her impressions and comments on the lesson observed, including adherence to the curriculum manuals, use of teaching skills, students' participation, and classroom atmosphere. Comments from instructor(s), for instance, on teaching materials, students' performance, and obstacles in conducting lessons, etc. were also recorded.

## RESULTS

Systematic classroom observation with the primary aim of rendering support to the instructors was conducted. Based on the analyses, several observations are highlighted. First, one of the salient findings indicated that all schools have incorporated the program (either 20- or 10-h program) wholly or partially into the formal curriculum, such as Life Education, Civic Education, Liberal Studies, and Integrated Humanities. Second, the average overall adherence to the curriculum manuals was 82.9% (range: 20–100%), which was quite high. In particular, only 5.4% of the observed units were rated below 50%.

Regarding the ratings for the quality of delivery, observers rated highly positive in the following areas: the quality of lesson preparation by the instructor(s), student interest, as well as student participation and involvement. In contrast, opportunity for reflection, use of positive and supportive feedback, and instructors' familiarity with the students were generally lower as compared to other dimensions of the program implementation quality. The findings on the quality of program implementation were summarized in Table 1. Reliability analysis showed that the scale was internally consistent (alpha = 0.91; mean interitem correlation = 0.47).

**TABLE 1**
**Summary of the Overall Ratings on the Units Observed in the Different Schools**

| | |
|---|---|
| **Total Number of Schools Observed** | 138 |
| **Total Number of Units Observed** | 221 |
| **Integration with School's Formal Curriculum** | |
| Life Education (%) | 67.9 |
| Civic Education (%) | 23.1 |
| Liberal Studies (%) | 4.1 |
| Integrated Humanities (%) | 3.6 |
| P.A.T.H.S. (%) | 0.9 |
| Moral Education (%) | 0.5 |
| **Overall Adherence (%)** | 82.9 |
| **Program Implementation Quality** | Mean* |
| 1. Student interest | 5.13 |
| 2. Student participation and involvement | 5.11 |
| 3. Classroom control | 4.97 |
| 4. Interactive delivery method | 4.88 |
| 5. Strategies to enhance student motivation | 4.85 |
| 6. Use of positive and supportive feedback | 4.60 |
| 7. Instructors' familiarity with the students | 4.68 |
| 8. Opportunity for reflection | 4.52 |
| 9. Degree of achievement of the objectives | 4.92 |
| 10. Time management | 4.99 |
| 11. Lesson preparation | 5.15 |
| 12. Overall implementation quality | 4.79 |
| 13. Success of implementation | 4.78 |

* Ratings on a 7-point scale with "7" denoting higher perceived level of the attribute.

For the qualitative comments made by the observer and the program implementers, they were analyzed using general qualitative analyses techniques[40]. Preliminary data analyses were conducted by the second author, and further checked by a colleague with a Doctoral degree to ensure the reliability in the interpretation process. The inter-rater reliability involving 20 randomly selected responses was 85%, which was relatively high. The authors also maintained conscious attitudes guarding against any personal biases and preoccupations that might influence the data interpretation. Based on the qualitative comments, factors conducive to successful implementation of the Tier 1 Program in the schools were identified, whereas factors that impeded the implementation quality were also explored. The related findings are presented in Tables 2 and 3, respectively.

## DISCUSSION

This paper reports the implementation quality of the Tier 1 Program (Secondary 1 Curriculum) delivered in the second year of the Full Implementation Phase of the P.A.T.H.S. Project. Utilizing an observation design, results from the current study largely support the findings of previous research and further confirm certain key features that contribute to the program effectiveness. First, the results support the idea of incorporating the program into the formal curriculum. As Shek and Sun[25] highlighted, incorporation

**TABLE 2**
**Factors Facilitating the Quality of Program Implementation**

| Aspect | Integrated Findings and Interpretations |
|---|---|
| Program | Diverse and interesting activities (N = 2) |
| People | Effective facilitation skills (N = 80) |
| | Active participation of students (N = 48) |
| | Motivated students (N = 26) |
| | Effective classroom management (N = 15) |
| | Good class atmosphere (N = 14) |
| | Good rapport with students (N = 11) |
| | Well-prepared lesson (N = 10) |
| Process | Interactive lesson (N = 9) |
| | Coteaching: social worker and teachers (N = 2) |
| Policy | Incorporated the program into formal curriculum (N = 2) |
| Place | Spacious venue (e.g., hall, activity room) (N = 2) |

**TABLE 3**
**Obstacles Impeding the Quality of Program Implementation**

| Aspect | Integrated Findings and Interpretations |
|---|---|
| Program | Perceived need to modify the materials (N = 1) |
| People | Unable to maintain class discipline (N = 27) |
| | Instructors lack teaching skills (N = 25) |
| | Some students are less motivated and involved (N = 23) |
| | Detached relationship between instructor(s) and students (N = 6) |
| | Uncommitted and/or inexperienced instructors (N = 5) |
| | Short attention span of students (N = 4) |
| | Some students have behavioral/learning problems (N = 2) |
| | Lack of time to prepare the lessons well due to heavy workload (N = 1) |
| Process | Poor time management (N = 6) |
| | Insufficient equipment in a classroom (e.g., computer, projector) (N = 2) |
| | Difficult to cater for students' needs through the suggested activities (N = 1) |
| Policy | NIL |
| Place | Poor design of the activity room (N = 1) |

of the Tier 1 Program into the formal curriculum is a viable strategy that could generate program success because the incorporation into the formal curriculum helps to avoid adding extra workload for the instructors. Second, it enables students to value learning.

Another salient finding is that the overall degree of adherence to the teaching units was high. This finding is consistent with the previous results[39], and it further disproves the myth that curricula-based positive youth development programs cannot be easily utilized and major modifications must be made for different adolescent populations. This conclusion is important because teachers and social workers usually believe the myth that the P.A.T.H.S. curriculum must be substantially modified before it can be used.

Another major conclusion is that different aspects of the program delivery were perceived to be very positive. These aspects include student interest and involvement (items 1 and 2) and quality of lesson preparation (item 11). These findings reflect teachers' diligence and their effort to prepare the lessons well and, so, students' interest and motivation were increased. This confirms that the key feature, "people", is a critical component of program success that is consistently mentioned in other studies[25] and further supported by the qualitative data in the present study, i.e., the comments by the observer.

Nevertheless, consistent with prior studies[39], the degree of reflection was the lowest among the items and this area deserves further attention. There are two plausible explanations. First, because of time constraints, teachers may not have had sufficient time to provide an opportunity for students to reflect. Second, the teaching style in Hong Kong is primarily didactic in nature and does not encourage such activity. Since reflection is a precious part of the learning process, it should be addressed in the training provided to the instructors before the implementation of the program.

In support of the previous findings[25], the qualitative data of the present study revealed that factors with reference to the 5"P"s (i.e., program, people, process, policy, and place) facilitated or impeded program implementation. To begin with, in terms of program ("program"), the perception of having adequate variety in the activity format was a determining factor that induced the instructors to deliver the program willingly, whereas perceived modification of teaching materials hinder the program implementation.

Results of the present study strongly suggest that instructors (i.e., teachers and/or social workers) ("people") play a very crucial role in the program implementation since they are the front-line workers of the project. The qualitative data obtained after a whole host of lesson observation offers a few glimpses of how to implement the program effectively in the classroom and, clearly, results pointed more to the importance of an effective teacher in the program implementation, including excellent preparation and design of a lesson, such as having a warm-up activity to motivate students, extremely good classroom management skills, effective teaching skills to motivate students to learn and to enhance student mastery of knowledge, etc. Other critical factors also include creating an open and positive classroom atmosphere, establishing a close rapport with students, and giving encouragement to students. Having committed and experienced instructors is also a crucial factor in order to facilitate the implementation of the program.

Apart from the instructors, the qualitative data also show that students play a significant role in program implementation, as the process of teaching and learning is reciprocal. The present findings indicated that students who are less motivated and passive in joining the activities frustrate the teachers and, simultaneously, affect the process of teaching and learning. A class with discipline problems is another area of concern, as it impedes the program implementation. This problem can be tackled by training teachers to have classroom management skills. Another feasible solution to discipline problems in the classroom is coteaching. Although the lesson could be run by one instructor per class, coteaching is highly recommended. First, the collaboration of teachers can lower the teacher-student ratio and the students' needs are better met, especially in the classroom where students with mixed abilities are prevalent. Second, the extra teacher can help to maintain class discipline and run activities, such as group work.

Pertaining to the implementation process ("process"), the qualitative data lend further support to the importance of possessing varied teaching skills that facilitate the implementation of the program. The use of multimedia teaching aids, interactive teaching strategies, facilitation skills, classroom management skills, and time management skills all help to create a positive learning atmosphere that raise the students' learning interest and motivation. Interactive teaching skills are particularly crucial as students' attention spans are relatively short. Their attention can only be captured when the lessons are lively or stimulating.

There are several impediments to the implementation of the program. As mentioned previously, classroom discipline is one of the major hindrances to the implementation of the program. Class disruptions severely affect teaching and learning and, so, classroom management skills are highly stressed. Another major hurdle is time management. Teachers felt pressured to complete all activities stipulated in the user's manual and activity handbook. However, the instructors are not encouraged to cut the activities when they perceive that they are running out of time, as it will seriously affect the students' learning[25]. Since time management is a crucial issue in the program implementation, it should be addressed in the training session provided to the instructors before they implement the program.

There are several limitations of the present study. First, since only one observer was involved in the class observation, the data collected might have biases that affect the credibility of the findings. However, as the "Co-Walker Scheme" aims to provide support to the instructors, the presence of two observers in the classroom might be seen as threatening. To validate the present findings, more observers, particularly those unrelated to the Project P.A.T.H.S., can be involved in the observation.

Second, consistent with the intrinsic problem of all observational studies where time sampling is involved, one needs to be conscious of the degree of generalizability of the present findings to other temporal and spatial contexts. One possible confounding factor is that the students may become more cooperative when there are visitors and outside observers. In addition, it is also possible that the instructors might be more motivated to teach well when being observed. Therefore, the use of ethnographic strategies with prolonged engagement and observations would be helpful. Third, despite the fact that the qualitative data have provided us with a substantial knowledge of program implementation in an authentic setting, i.e., classroom, the data collected are solely from one observer and bias might be associated with those comments[41]. This limits the generalizability of results and, therefore, data must be interpreted with caution. As such, more structured and systematic data should be collected in future to validate the results. Despite these limitations, the present findings lend further support to the successful implementation of the Tier 1 Program of the Project P.A.T.H.S. in schools.

## ACKNOWLEDGMENTS

The preparation for this paper and the Project P.A.T.H.S. were financially supported by The Hong Kong Jockey Club Charities Trust.
